# Effect of cyanocobalamin (vitamin B12) on paraquat-induced brain injury in mice

**DOI:** 10.22038/IJBMS.2022.64164.14128

**Published:** 2022-06

**Authors:** Marzieh Jafari Fakhrabad, Mohammad Moshiri, Fatemeh Ariakia, Vahid Reza Askari, Zahra Salmasi, Leila Etemad

**Affiliations:** 1 Department of Toxicology, School of Pharmacy, Mashhad University of Medical Sciences, Mashhad, Iran; 2 Medical Toxicology Research Center, School of Medicine, Mashhad University of Medical Sciences, Mashhad, Iran; 3 Department of Clinical Toxicology, Imam Reza Hospital, Mashhad University of Medical Sciences, Mashhad, Iran; 4 Pharmaceutical Research Center, Pharmaceutical Technology Institute, Mashhad University of Medical Sciences, Mashhad, Iran; 5 Applied Biomedical Research Center, Mashhad University of Medical Sciences, Mashhad, Iran; 6 Department of Pharmaceutical Sciences in Persian Medicine, School of Persian and Complementary Medicine, Mashhad University of Medical Sciences, Mashhad, Iran; 7 Department of Persian Medicine, School of Persian and Complementary Medicine, Mashhad University of Medical Sciences, Mashhad, Iran; 8 Nanotechnology Research Center, Pharmaceutical Technology Institute, Mashhad University of Medical Sciences, Mashhad, Iran; 9 Department of Pharmaceutical Nanotechnology, School of Pharmacy, Mashhad University of Medical Sciences, Mashhad, Iran; 10 Department of Pharmaceutical and Food Control, School of Pharmacy, Mashhad University of Medical Sciences, Mashhad, Iran

**Keywords:** Apoptosis, Inflammation, Neurotoxicity, Oxidative stress, Paraquat, Vitamin B12

## Abstract

**Objective(s):**

The goal of this study was to evaluate the neuroprotective effects of vit B12 on paraquat-induced neurotoxicity.

**Materials and Methods:**

Thirty-six male mice were randomly divided into six groups. Three groups were treated intraperitoneally with paraquat (10 mg/kg) twice a week (with a 3-day interval) for 3 weeks. Normal saline, vit B12 (1 mg /kg), or vit C (50 mg/kg) was injected 30 min before paraquat administration. Other groups only received normal saline (control), vit B12, or vit C in the same protocol. Motor performance and coordination were assayed by challenging beam traversal, pole, open field, and rotarod tests. The hippocampus and serum samples were isolated to evaluate the oxidative stress (GSH and ROS), apoptosis (caspase 3), and inflammatory markers (TNF-α and IL-1β).

**Results:**

Administration of paraquat leads to induction of motor deficits, which were improved by treatment with vit B12. In addition, vit B12 could prevent oxidative damage, apoptosis, and inflammation caused by paraquat.

**Conclusion:**

It seems that vit B12 could be a novel therapeutic agent in the management of paraquat induced-neurotoxicity.

## Introduction

 Paraquat (PQ) dichloride (1,1_ dimethyl-4–4_-bipyridylium dichloride) is a herbicide with specific use in the control of weeds and grass(1). Accidental or intentional PQ poisoning is considered a major health problem (1, 2) and identified as category 2 in the acute toxicity hazard class (3). PQ is absorbed through the gastrointestinal tract, respiratory system, and skin, however, oral absorption is the most common route of poisoning (1, 4). The high fatality rate of PQ poisoning is due to multiple organ dysfunction ranging from impairment in the lung, gastrointestinal tract, pancreas, kidney, liver, and heart to the brain(1, 4-6). The lung is the main target organ in PQ poisoning, and respiratory failure is the most common cause of death. The clinical manifestation of PQ acute poisoning is usually dose-dependent, and classified into three stages: mild, moderate, and fulminant (1, 7-9). Epidemiological studies indicate that chronic exposure to PQ can be a risk factor for neurological complications and neurodegenerative diseases such as seizures, coma, and Alzheimer’s, Parkinson’s, and Huntington’s disease (5, 10-14). PQ has a similar structure to MPP ^+^ (1-methyl-4-phenylpyridinium ion), which is the active metabolite of MPTP. MPTP is a neurotoxic compound that induces Parkinson’s disease (PD)(15, 16). *In vitro* and *in vivo* toxicological studies, as well as human reports, have shown a significant decrease in the number of dopaminergic neurons in three regions of the brain: substantia nigra (SN), hippocampus, and frontal cortex, after long-term exposure to PQ (13, 17-19). PQ can gradually pass the blood-brain barrier (BBB) by neutral amino acid transporters and reach neurotoxic levels, which in turn promotes neurological complications (20). PQ induces excessive generation of reactive oxygen species (ROS) and nitrite species that can lead to cellular damage through activation of inflammatory factors and NF‐κB, lipid peroxidation, mitochondrial dysfunction, and apoptosis (21, 22). Up to now, no medication has been approved by the U.S. Food and Drug Administration (FDA) for PQ poisoning and treatment. 

Cobalamin (vit B12), plays a pivotal role in metabolic processes and is required for optimal function of the CNS and production of red blood cells (23). In mammalian cells, the activity of two important enzymes, methionine synthase (MS) and methyl malonyl CoA mutase (MCM) is dependent on vit B12. Vit B12 deficiency is associated with reduction of enzyme activity and therefore decreased synthesis of phospholipids and myelin and increased neurological damage (24-28). Vit B12 deficiency along with raised homocysteine and methylmalonic acid levels have been observed in patients with Alzheimer’s and PD (26, 27, 29, 30). It reduces or inhibits the synthesis of inflammatory mediators such as TNF-α, IL-6, and hs-CRP and increases the antioxidant enzymes such as SOD (superoxide dismutase), CAT(catalase), and GPx (glutathione peroxidase)(31-34). Studies have shown that B vitamins can prevent the nervous system dysfunction through scavenging the ROS, especially superoxide radicals, and reduction of inflammatory markers (35, 36). Vit B12 can penetrate the BBB and exerts its neuroprotective effects in neurodegenerative diseases (37, 38). It has a functional role in the peripheral nervous system repairing and reducing the destruction of nerve cells as well as maintaining the balance between neurotoxic and neurotrophic factors in the region of the hippocampus and cerebral cortex (30, 39, 40). This study aimed to investigate the neuroprotective effects of vit B12 on PQ neurological complications by inspecting changes in behavioral responses, inflammatory mediators, the oxidant-antioxidant system as well as apoptosis markers.

## Materials and Methods

***Reagents***

PQ (active ingredient content of 200 g/L Arad chem, China), vit B12(Excir company, Iran), vit C (Latif Company, Iran), Ellman’s reagent (DTNB, Sigma Aldrich, German), 2,7-dichloro dihydro fluorescein diacetate (DCFH-DA, Sigma), TNF-α(mouse) ELISA kit (88-7324- Invitrogen, Inc), IL-1β(mouse) ELISA kit (88-7013-Invitrogen, Inc), caspase-3(Cell Signaling, #9665), horseradish-peroxidase conjugated anti-rabbit antibody (Cell Signaling, #7074).

***Animal treatments***

Male albino mice (aged 7 weeks, weighing 25-30 gr) were purchased from the animal room of the School of Pharmacy, Mashhad University of Medical Sciences (Mashhad, Iran). All animals were kept at constant room temperature (21±2 ^°^C) under a 12/12 hr light/dark cycle at least 10 days prior to the test. 36 male mice were randomly divided into six groups of six each. Three groups were treated intraperitoneally with PQ (10 mg/kg) twice a week (with a 3-day interval) for 3 weeks (41). Normal saline, vit B12 (1 mg/kg), or vit C (50mg/kg) was injected 30 min before PQ administration. Other groups received normal saline alone (control) or plus vit B12 or vit C, in the same protocol. 

The body weight changes of animals were evaluated every day of treatment during the experiment. Behavioral tests were performed three days after the last injection. At the end of the procedure, the animals were sacrificed and the midbrain was also removed and stored at -80 ^°^C for further analysis. 

***Motor function assessment***

*Challenging beam traversal*

Briefly, the apparatus was made of a Plexiglas beam consisting of four segments (25 cm each, 1 m total length), the width of each section was different from the widest to the narrowest section (3.5 cm to 0.5 cm by 1 cm increments). Animals were placed on the beam and allowed to traverse the beam from the widest to the narrowest end, which leads directly into the home cage. On the day of the test, a metal mesh grid (1 cm^2^) corresponding to width of each segment was placed on top of the beam. Animals of each group were individually videotaped while traversing the metal grid-surfaced beam for a total of 5 trials, the error rate per step, number of steps, and time to traverse the beam were assessed. The experiment was performed from 7:00 AM to 9:00 AM.

*Pole test*

 The pole test is a simple behavior test used to assess motor performance and coordination. In summary, mice were placed head up on the top of a vertical wooden pole apparatus of 50 cm height (diameter: 1cm). Animals were habituated for 2 consecutive days before the experiment. The time to orient down (T-Turn), time for arriving at the 25 cm points (T-25cm), and climb down (T-Total) were recorded. The test was performed from 10:00 AM to 12:00 PM.

*Open field test*

The open-field test is used to measure locomotor activity, anxiety, and exploratory behavior. The open field apparatus consisted of white wood 60×60 cm with a 40 cm high wall, the floor was divided by blue lines into 25 squares. The mice were trained one day before the test for 5 min. Briefly, each mouse was individually placed in the center of the open field, and locomotor activities were evaluated for 5 min. The behavioral parameters registered were the total number of squares crossed (frequency with which the mouse crosses the squares with all four paws), the number of central squares crossed, and the number of outer squares crossed; the three measures were referred to as total, central, and peripheral locomotion, respectively. The floor was cleaned between each mouse with 20% ethanol. The test was performed from 1:00 PM to 3:00 PM.

*Rotarod test*

Motor coordination and balance were assessed using the rotarod test. The rotarod apparatus consists of a metal rod coated with rubber rotating on an axis. Briefly, mice were trained for one day on a rod rotating at the initial and final speeds of 10 and 20 rpm (the acceleration time was 20 sec). On the next day, mice were placed on the rotating rod for a maximum period of 300 sec. The time-to-fall off the Rotarod was recorded. The experiment was performed from 4:00 PM to 6:00 PM

***Oxidative stress assays***

*Glutathione assay*

In order to measure the content of glutathione (GSH), the midbrain was removed and 10% tissue homogenates were mixed with an equal amount of 10% trichloroacetic acid. After centrifugation (5000 rpm, 4 ^°^C, 10 min), Ellman’s regent in PBS (phosphate buffer saline, pH=8 ) was added to the supernatant. Finally, the change of absorbance was measured at 412 nm by a spectrophotometer. The total GSH content was estimated using the standard curve.

*ROS assay*

ROS production in the brain was assayed using the fluorogenic dye Dihydrodichlorofluorescein diacetate (H2DCFDA). H2DCFDA can penetrate the cell and convert to a non-fluorescent compound by intracellular esterase, and then to a highly fluorescent compound 2,7-Dichlorofluorescein (DCF) through oxidation. In this method, briefly, midbrain samples were homogenized at room temperature (RT) in 2 ml phosphate buffer (PH: 7.4). After centrifugation (1000 rpm, 4 ^°^C, 10 min), 15 µl of DCFH-DA regent (5µM) was added to the supernatant. The conversion of DCFH-DA to the fluorescent product DCF was measured using a spectrofluorimeter with excitation at 484 nm and emission at 530 nm. ROS formation was quantified using an H_2_O_2_-standard curve. 

***Apoptotic assay***

Western blotting was carried out to assess the caspase-3 expression level. In summary, the midbrain samples were added to RIPA-lysis buffer (50 mM Tris-HCl(pH=7.4), 150 mM NaCl (1%), Triton X-100 (1%), Sodium Deoxycholate (0.1%), SDS, Sodium Orthovanadate, Sodium Fluoride, EDTA,1 mM PMSF and protease inhibitor cocktail) on ice and mechanically homogenized. After centrifugation (10000 rpm, for 10 min, at 4 ^°^C), the protein content was determined using the Bradford method with bovine serum albumin as standard. Equal amounts of protein extracts were loaded on 10% SDS-PAGE and separated by electrophoresis. In the next step, proteins were transferred onto the polyvinylidene difluoride (PVDF) membrane and blocked with 5% skim milk for 2 hr at RT. Then, the blots were incubated with specific primary antibody caspase-3 at 4 ^°^C overnight. Each membrane was washed three times, and then the blots were subsequently incubated with secondary antibody HRP for 1.5 hr at 37 ^°^C. Enhanced chemiluminescence (Pierce ECL western blotting substrate) and Alliance gel doc (Alliance 4.7 Gel doc, UK) were used for detection of the protein bands.

***Inflammatory factors assay***

TNF-α and IL-1β are potent inflammatory cytokines produced by macrophages/monocytes in response to inflammation. Midbrain levels of TNF-α and IL-1β were measured using an ELISA kit in accordance with the manufacturer’s protocol. The absorbance was measured at 450 nm.

***Statistical analysis***

All data were expressed as mean±SD (n=6). The statistical significance of differences between the means was determined using one-way ANOVA, followed by Tukey–Kramer test using SPSS (ver. 16.0) software. The level of significance was set at *P*<0.05.

## Results

***Body Weight***

The result showed that there was no difference in the weight changes of animals between different groups (data not shown). The rate of mortality was zero.

***Motor function analysis***

*Challenging beam traversal test*

**Figure 1:**
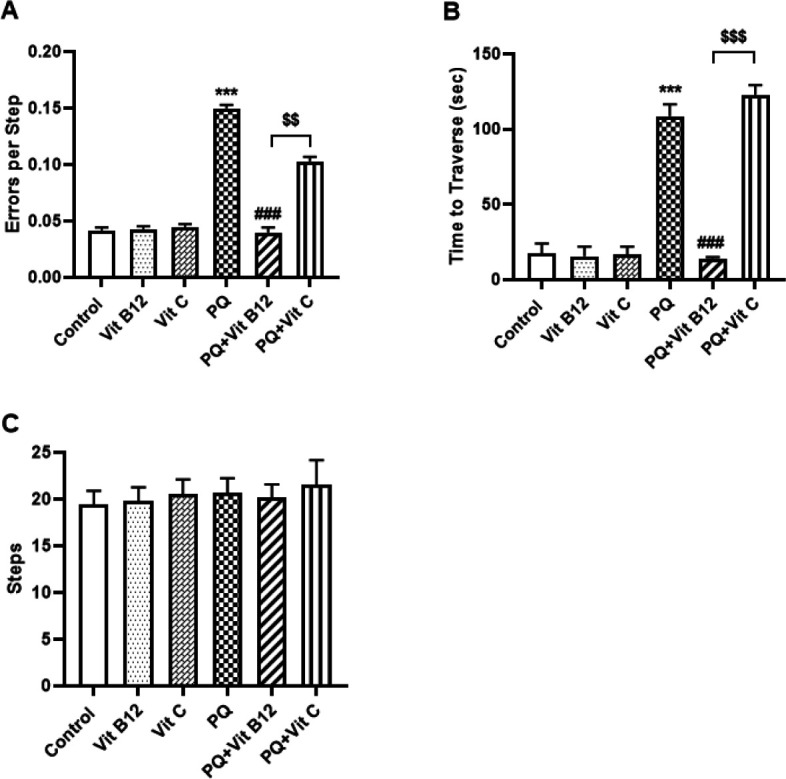
Motor impairment and coordination were measured in PQ mice with a challenging beam traversal test. Errors per step(A), time to traverse (B), and the number of steps (c) were measured three days after the last injection. PQ, vit B12, and vit C were injected intraperitoneally (IP) at 10, 1, and 50 mg /kg, respectively. PQ was administered twice a week (with a 3-day interval) and vit B12, vit C, or normal saline was administered 30 min before PQ administration. The results indicated that PQ mice made more errors and took a long time to traverse the beam compared with the control group. In contrast, Vit B12 improved PQ-induced motor deficits. Results were presented as mean±SD (n=6). ***P<0.001 versus control, ^###^*P<*0.001 compared with PQ group, ^$$^*P<*0.01 and ^$$$^*P<*0.001 versus PQ+ Vit C

In this study, for assessment of fine motor performance and coordination, animals underwent the challenging beam test. The results indicated that PQ-treated mice made significantly more errors per step compared with the control mice (*P*<0.001)( Figure 1A). *Post hoc* analysis also revealed that the PQ group took a significantly longer time to traverse the beam (*P*<0.001)( Figure 1B). While no difference was observed in the number of steps between groups (Figure 1C). Pretreatment with vit B12 reversed the toxic effects of PQ. The results showed that pretreatment with vit B12 significantly reduced the number of errors per step (*P*<0.001) and time to traverse the beam (*P*<0.001) compared with the PQ-treated group. In addition, vit B12 treatment significantly improved the errors per step (*P*<0.01) and time to traverse the beam (*P*<0.001 ( in PQ-treated mice compared with the PQ+vit C group.

*Pole test*

**Figure 2:**
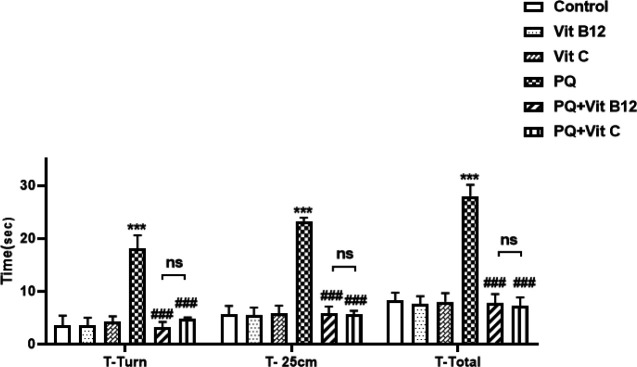
Effect of vit B12 on PQ-induced impaired behavioral performance in the pole test. The time to orient down (t-turn) and time for arriving at 25 cm (T-25cm) and climb down (T-Total) points were recorded three days after the last injection. PQ, vit B12, and vit C were injected intraperitoneally (IP) at 10, 1, and 50 mg/kg, respectively. PQ was administered twice a week (with a 3-day interval) and vit B12, vit C, or normal saline was administered 30 min before PQ administration. Results were expressed as the mean±SD(n=6). ***P<0.001 versus control, ^###^*P<*0.001 compared with PQ group. ns= no significant difference

Analysis of pole test performance demonstrated markedly increased T-Turn, T-25cm, and T-Total in the PQ group compared with the control group (*P*<0.001) (Figure 2). While pretreatment with vit B12 or vit C improved the PQ-induced motor deficits. The data showed that treatment with vit B12 significantly decreased T-Turn, T-25cm, and T-Total as compared with the PQ group (*P*<0.001). No difference was observed between vit B12 and vit C treatment groups.

*Open field test*

**Figure 3:**
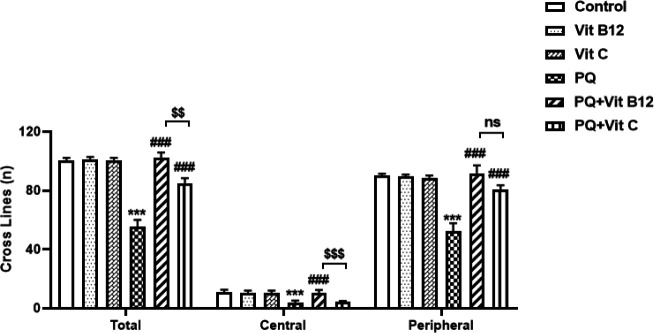
Effect of treatments on the motor behavioral activities in the open field test in mice. PQ, vit B12, and vit C were injected intraperitoneally (IP) at 10, 1, and 50 mg/kg, respectively. PQ was administered twice a week (with a 3-day interval) and vit B12, vit C, or normal saline was administered 30 min before PQ administration. Results were expressed as the mean±SD (n=6). ***P<0.001 PQ versus control, ^###^*P<*0.001 compared with PQ group, ^$$^*P<*0.01 and ^$$^*P<*0.001 PQ+Vit B12 versus PQ+vit C. No significant (ns) difference between vit B12 and vit C treatment at *P˃*0.05

The animals were assessed for motor behavioral activity after the last treatment using the open field test. Our findings suggested that PQ decreased the open field parameters. *Post hoc* analysis revealed a significant reduction in the total distance traveled, number of entries into the center zone, and peripheral locomotion by PQ-treated mice when compared with the control group (*P*<0.001). However, pretreatment with vit B12 markedly reversed the PQ-induced motor dysfunction (*P*<0.001) (Figure 3). Administration of vit C also significantly increased the total distance traveled and peripheral locomotion (*P*<0.001), while no difference was observed in the number of central locomotion as compared with the PQ group. Data analysis also revealed that vit B12 treatment increased the open field parameters (the total distance traveled and central locomotion) compared with the PQ+ vit C group (respectively; *P*<0.01 and *P*<0.001).

*Rotarod test*

**Figure 4.**
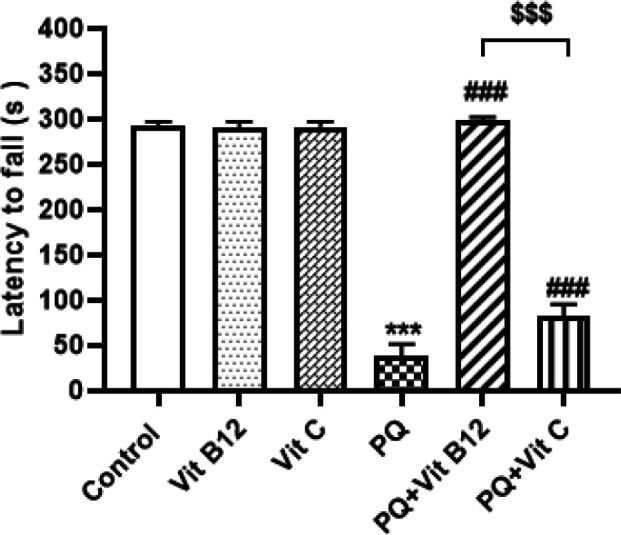
Effect of PQ, vit B12, and vit C on motor coordination in Rotarod test. PQ, vit B12, and vit C were injected intraperitoneally (IP) at 10, 1, and 50 mg/kg, respectively. PQ was administered twice a week (with a 3-day interval) and vit B12, vit C, or normal saline was administered 30 min before PQ administration. Results were expressed as the mean ± SD (n=6). ***P<0.001 versus control , ^###^*P<*0.001 compared with PQ group and ^$$$^*P<*0.001 versus PQ+vit C

The results obtained showed that repeated administration of PQ decreased motion balance in the rotarod test. The results indicated that PQ-treated mice had markedly reduced latency to fall when compared with the control group (*P*<0.001). While pretreatment with vit B12 and vit C significantly increased the retention time on the rotating rod as compared with the PQ group (*P*<0.001). In addition, vit B12 treatment increased the latency of falls compared with the PQ+ vit C group (*P*<0.001) (Figure 4). 

***Effects of Vit B12 on GSH levels in PQ-intoxicated mice***

**Figure 5:**
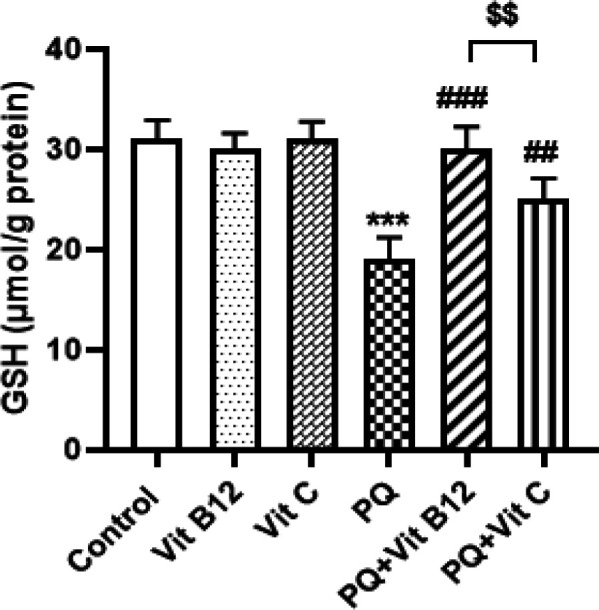
Effect of vit B12 on reduced glutathione concentration in the midbrain of PQ-intoxicated mice. PQ, vit B12, and vit C were injected intraperitoneally (IP) at 10, 1, and 50 mg /kg, respectively. PQ was administered twice a week (with a 3-day interval) and vit B12, vit C, or normal saline was administered 30 min before PQ administration. Analysis of the results showed that PQ affects GSH levels in the midbrain. Levels of GSH in PQ-treated mice were significantly reduced compared with the control group. However, treatment with vit B12 and vit C, significantly increased GSH levels when compared with the PQ-treated mice. Results were expressed as the mean±SD (n=6). ***P<0.001 versus control ,^##^*P<*0.01, ^###^*P<*0.001 compared with PQ group and ^$$^*P<*0.01 versus PQ+vit C

Based on the results of the present study, PQ significantly reduced levels of GSH in the midbrain as compared with the control group (*P*<0.001). However treatment with vit B12 (1 mg/kg) or vit C (50 mg/kg), significantly increased the GSH levels within the midbrain as compared with the PQ-treated mice (respectively; *P*<0.001, *P*<0.01), which means that vit B12 and vit C could have a protective effect against oxidative stress caused by PQ (Figure 5). In addition, vit B12 treatment significantly increased the GSH levels in PQ-treated mice compared with the PQ+ vit C group (*P*<0.01).

***Effect of Vit B12 on PQ-induced oxidative stress in the midbrain***

**Figure 6:**
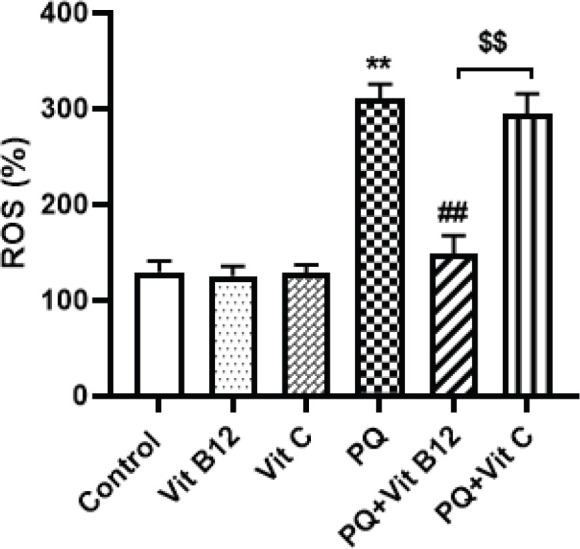
Effect of B12 treatment on PQ-induced ROS generation in the midbrain. PQ, vit B12, and vit C were injected intraperitoneally (IP) at 10, 1, and 50 mg /kg, respectively. PQ was administered twice a week (with a 3-day interval) and vit B12, vit C, or normal saline was administered 30 min before PQ administration. The results showed that vit B12 could prevent increased PQ-induced ROS. Results were expressed as the mean±SD (n=6). **P<0.01 versus control , ^##^*P<*0.01 compared with PQ group and ^$$^*P<*0.01 versus PQ+vit C

To determine the role of ROS in PQ-induced oxidative stress and nerve damage, the levels of ROS were evaluated using DCFH-DA fluorescence staining in the midbrain. The results of the current study indicated that the levels of ROS in the midbrain PQ-treated mice were significantly enhanced compared with the control group (*P*<0.01) (Figure 6). However, pretreatment with vit B12 (1mg/kg) significantly decreased the level of ROS compared with PQ treated group (*P*<0.01). Analysis of the results showed that vit B12 could reduce ROS levels and had a protective effect against PQ-induced oxidative stress, while no difference in vit C was observed in the ROS levels compared with the PQ group. Vit B12 treatment also markedly prevented excess ROS generation by PQ compared with the PQ+ vit C group (*P*<0.01).

***Effect of Vit B12 on PQ-induced apoptosis***

**Figure 7:**
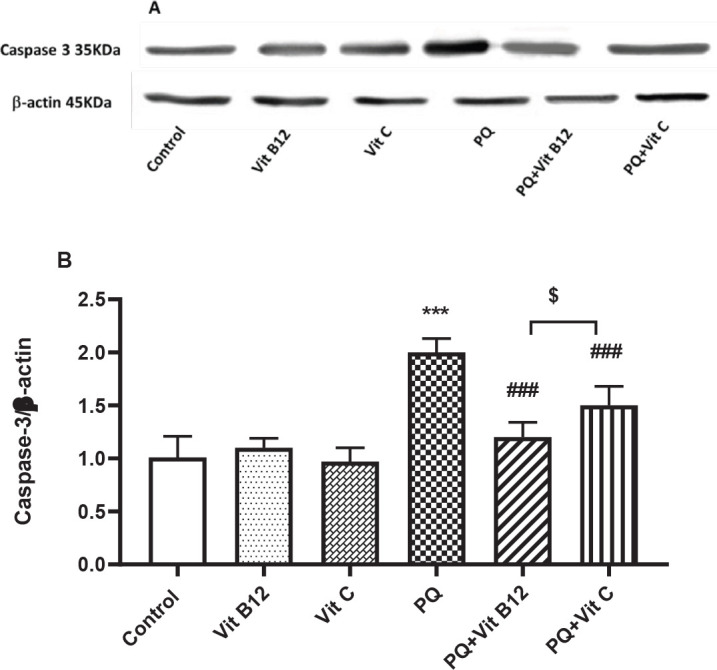
Effect of vit B12 on the level of caspase 3 in the midbrain of PQ-intoxicated mice. PQ, vit B12, and vit C were injected intraperitoneally (IP) at 10, 1, and 50 mg/kg, respectively. PQ was administered twice a week (with a 3-day interval) and vit B12, vit C, or normal saline was administered 30 min before PQ administration. (A) Representative photograph of the western blot analysis. (B) Densitometric data of protein analysis. Values were presented as the mean±SD (n=6). ***P<0.001 PQ versus control, ^###^*P<*0.001 compared with PQ group, and ^$^*P<*0.05 versus PQ+vit C

PQ exposure up-regulated expression of caspase-3 protein level in the midbrain of intoxicated mice compared with the control (*P*<0.001). Although vit B12 and vit C treatment resulted in decreased apoptosis marker level in comparison with the PQ group (*P*<0.001), only vit B12 could return it to the normal level. A significant difference was observed between PQ+ vit B12 and PQ+ vit C groups (*P*<0.05) (Figures 7 A and B).

***Effect of Vit B12 on PQ-induced inflammation***

**Figure 8:**
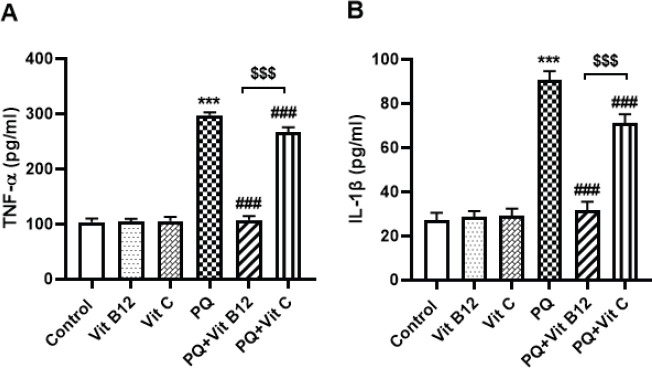
Effect of vit B12 on the midbrain level of TNF-α and IL-1β in PQ-intoxicated mice. PQ, vit B12, and vit C were injected intraperitoneally (IP) at 10, 1, and 50 mg/kg, respectively. PQ was administered twice a week (with a 3-day interval) and vit B12, vit C, or normal saline was administered 30 min before PQ administration. Values were presented as the mean±SD (n=6). ***P<0.001 PQ versus control, ^###^*P<*0.001 compared with PQ group and ^$$$^*P<*0.001 versus PQ+vit C

Analysis of the results revealed that administration of PQ led to a significant increase in the concentration of TNF-α and IL-1β as compared with the control group) *P*<0.001). However, pretreatment with vit B12 efficiently decreased the level of TNF-α and IL-1B as compared with the PQ group (*P*<0.001). Vit C treatment also significantly reduced the level of TNF-α and IL-1B (*P*<0.001), which means that vit B12 and vit C could have a protective effect against PQ-induced inflammation. However, there was a significant difference between vit B12 and vit C (*P*<0.001)(Figures 8 A and B).

## Discussion

The results of the present study revealed that vit B12 can decrease the PQ-induced behavioral and neurochemical alterations. PQ is an herbicide for the control of weeds with a broad spectrum of toxicological properties for humans. Epidemiological studies indicated that chronic exposure to PQ can be a risk factor for neurodegenerative diseases such as Parkinson’s, Alzheimer’s, and Huntington’s (5, 18, 42-45)**. **Studies with rodent models have suggested that intraperitoneal (IP) administration of PQ induced motor impairment as well as loss of hippocampal neurons, leading to learning and memory deficits (27)**. **The results of this study showed that repeated injection of PQ (10 mg/kg) induced motor impairment and incoordination in pole, challenging beam traversal, and rotarod tests as well as a decrease in spontaneous motor activity in the open field test. In this study, administration of PQ increased the time to orient down (T-Turn) and climb down (T-Total), as a measure of motor impairments, which was in agreement with the results of previous studies (46-48). *In vivo* studies showed that chronic exposure to PQ induced significant impairments in balance and vertical limb movements through prolonged latency of T25cm, T-turn, and T-total in the pole test (41, 49, 50). The effect of PQ on motor performance and coordination was also assessed in the challenging beam traversal test. Our findings were in line with the study conducted by Fernagut *et al*. PQ administration (10 mg/kg, IP) prolonged the time to traverse the beam and increased the number of errors in challenging beam traversal tests as an indicator for motor deficits and muscle incoordination (51). PQ administration also remarkably reduced the spontaneous motor activity and the parameters evaluated in the open field and confirmed the neurobehavioral disorders (52-54). It was documented that administration of PQ (10 mg/kg, twice a week for 3 consecutive weeks) induced a decrement in the number of line crossings in the open field test (55, 56). It was also reported that subcutaneous injection of PQ at various doses (5,10, and 20 mg/kg) significantly decreased the total distance traveled time and ambulatory counts as well as stereotyped behavioral activities in PQ-treated rats in the open field test (57). 

 In addition, PQ treated mice showed lower latency to fall and motor incoordination in the rotarod test. The rotarod test is widely used to assess motor coordination and balance and provides one measure of locomotor activity. It is well documented that PQ injection at 10 mg/kg can induce deficits in motor balance, muscle weakness, and reduce latency to fall on the rotarod task (44, 54). Prenatal exposure to PQ and sensorimotor deficits and decline in latency to fall in rotarod performance in mice offspring confirmed the existence of neuromuscular incoordination because of modification in cerebellar function (58-60). 

However, vit B12 treatments clearly improved PQ-induced movement deficits evaluated in behavioral tests. Cyanocobalamin (CNCbl,vit B12) is a crucial micronutrient in numerous biological processes with the optimal function of the neural system (61). It has been reported that vitamin B inhibits the release of excessive glutamate and excitotoxic neurotransmitters in the brain (62, 63). Vit B12 can penetrate the BBB, which is evidence of support for its neuroprotective effects in neurodegenerative diseases(37, 38)**. **It showed a neuroprotective effect in the tibial nerve-damaged model in rats through improvement of motor function and attenuation of the neuron cell degeneration (64). Moreover, vit B12 on sciatic nerve damage reduces the destruction process and improves its regeneration (37). 

In line with our results, vit B12 (1 mg/kg) reduced motor impairment in a dose-dependent manner in a rat model of multiple sclerosis and ameliorated the open-field behavior (65). Vit B12 deficiency in rat diet also led to reduction of line distance traveled and motor activity as well as motor balance impairment and a decline in the latency of falls on the open field and rotarod tests (66, 67). It was documented that dietary supplementation containing vit B12 and folate, in a model of ischemic injury in mice, could improve the motor activity in the rotarod test (68). Vitamin C also known as ascorbic acid (AA) is a water-soluble vitamin that plays a vital role as an antioxidant in reducing oxidative stress and inflammation and as a co-factor in many major biological processes (69, 70). Although vit C is not able to directly penetrate the BBB, its oxidized form (dehydroascorbic acid) easily enters the brain via glucose transporters, which is evidence of amplification of its antioxidant potential in CNS (71, 72). In an experimental sepsis model in rats, vit C administration at a high dose (200 mg/kg, IP) effectively improved cognitive impairment in the Morris Water Maze test (73). Another study showed vit C deficiency in mice diet also led to behavioral disorders and reduced locomotor activity that was alleviated by vit C treatment (74). In a recent study, the results of pole, open field, and rotarod tests showed the protective effect of vit C against PQ-induced behavioral alterations. However, challenging beam traversal test results did not confirm it**.** Indeed, it was indicated that vit B12 exerted its neuroprotective effect stronger than vit C.

Different methods such as inflammatory and oxidative stress have been proposed in PQ neurotoxicity up to now (75, 76). In the present study, repeated administration of PQ also impaired prooxidative/antioxidative homeostasis through excessive generation of ROS and reduction in GSH content in the midbrain. *In vivo* and *in vitro* studies confirmed that PQ induced neurotoxicity and oxidative stress via overproduction of ROS and decrease in GSH and antioxidant enzyme levels (77-79). Another study revealed frequent injections twice a week for 6 weeks of PQ (10 mg/kg) and maneb (MB) (30 mg/kg) considerably activated ROS production and decreased GSH levels in the midbrain of mice. It was also documented that administration of PQ significantly reduced total GSH content in three regions of the brain: striatum, hippocampus, and cortex in mice (80). PQ-induced decreased GSH content and increased ROS levels were also reported in the substantia nigra pars compacta (SNpc) and midbrain regions of the mice (81-82)**.** Excessive accumulation of ROS in the brain can damage a wide range of cellular compounds including DNA, proteins, and lipids, and has been implicated as a major underlying cause of neuropathy and various disorders including neurodegenerative diseases such as PD (21, 83-85). The result of this study clearly showed excess ROS generation and reduced GSH content by PQ administration in the midbrain that was returned to the normal rates by vit B12 treatment. Research has shown that vit B12 plays a protective role against elevated superoxide levels and preserves GSH content in the reduced state (86, 87)**.** In our previous study, vit B12 administration could prevent the neurotoxicity of methamphetamine in the striatum and cortex regions of the mouse brain via an increase in GSH level and decreased apoptotic index (88). Moreover, vit B12 therapy after brain ischemia significantly increased the expression of the anti-oxidant enzyme (GSH and SOD( and inhibited lipid peroxidation in the brain of ischemic rats which was in harmony with our study (89). In the recent study, the protective effect of vit C against PQ-induced oxidative stress was only shown in increased GSH content and not in ROS levels. The antioxidant effect of vit c through decreasing the ROS level and increasing the GSH level has been shown in different studies (90). In contrast, it was indicated that the vit C treatment in PQ-induced lung toxicity can either accelerate the HO^·^ production or exacerbate oxidative stress (91). 

 In our study, the significant elevation of midbrain TNF-α and IL-1β levels in mice treated with PQ alone was observed, which was significantly attenuated by vit B12 administration.

Inflammatory processes have been confirmed to play a vital role in neurodegenerative disorders such as Alzheimer’s and PD (92). It has been reported that frequent exposure to PQ caused an induction in proinflammatory cytokine gene expression which led to increased inflammatory-related genes such as IL-6, IL-1β, and TNF-α in three regions of the brain: SN, hippocampus, and frontal cortex (17, 93-95). In line with our study, subchronic administration of PQ significantly elevated the amount of TNF-α in the midbrain and induced parkinsonism in mice (96, 97). However, vit B12 pretreatment attenuated PQ-induced inflammatory response by reducing TNF-α and IL-1β levels. The result of clinical study of intensive care unit patients also indicated that high-dose parenteral vit B12 could modulate systemic inflammation (98). It seems that vit B12 can be useful as treatment for a broad spectrum of inflammatory diseases associated with oxidative stress (99-101). Human studies reported a considerable imbalance between serum vit B12 and inflammatory mediator (TNF-α and IL-1β) levels, which could recover by vit B12 treatments (27, 102). In an experimental allergic encephalomyelitis model in rats, co-administration of bee venom and vit B12 reduced TNF-α level, gliosis, and NO generation (106). An *in vitro* study conducted by Li *et al*. showed that vit C treatment significantly inhibited the expression of inflammatory factors (IL-1β, and IL-6) caused by hypoxia in rat intestinal epithelial cell line (IEC-6) (104). In contrast, a clinical study in 25 subjects with metabolic syndrome showed intravenous vit C administration increased the expression of inflammatory-related genes such as TNF-α, Interleukin 4 (IL-4), and Interferon-gamma (IFN-γ) in the mononuclear cells (105). Our results showed that vit C treatment significantly reduced midbrain inflammatory factors (TNF-α, IL-1β) which was consistent with previous studies (106-108). However, the results revealed that vit B12 is more effective than vit C in reduction of PQ-induced inflammatory cytokines.

We also examined the effects of vit B12 on the expression of apoptosis markers in the midbrain of mice treated with PQ. In accordance with previous studies, the apoptosis process was considered a cause of PQ toxicity (109). It has been reported that acute exposure to PQ (40 mg/kg, twice daily) significantly increased the expression of caspase 3 and apoptotic cells in the brain rat (110). Another study indicated that PQ administration significantly elevated caspase-3 levels and cell apoptosis in the hippocampus and midbrain sections of intoxicated mice (111, 112). Vit B12 prevented the apoptosis induced by PQ with strong down-regulation of caspase 3 in the midbrain, which often serves as a trigger for apoptosis cascades. In a pneumoniae meningitis rat model, administration of vit B12 could modulate caspase 3 activity and apoptosis in the hippocampal dentate gyrus (113). The findings of animal studies showed that treatment with a B12 supplement effectively inhibits the process of apoptosis via decreased mRNA expression levels of caspase 3 and 8 in the abdominal aorta or brain in rats (47, 114). It was also reported that pretreatment of vit C (250 mg/kg) significantly reduced caspase-3 activity and prevented apoptotic neurodegeneration caused by pentylenetetrazol (PTZ) in rat brains (115). In the present study, vit B12 significantly reduced the PQ-induced caspase 3 levels in the midbrain, in comparison with vit C.

## Conclusion

Finally, the present results indicate that vit B12 had potential preventive and therapeutic effects on PQ-induced neurotoxicity and locomotor activity impairment in mice. One explanation could be that vit B12 exerted direct antioxidant and free radical scavenging activities. Vit B12 also possessed noteworthy anti-inflammatory properties and decreased the level of IL-1β and TNF-α. Vit B12’s protective effect resulted in attenuation of apoptosis cell death through inhibiting expression of caspase 3. These findings suggest that Vit B12 can be a potentially novel therapeutic strategy for prevention of PQ-induced neurotoxicity.

## Authors’ Contributions

LE and MJF conceived the original idea. MJF and FA performed experiments. LE and VRA supervised the research. LE, MJF, ZS, VRA, and MM analyzed the data. MJF, ZS, LE, and FA prepared the original draft. MJF, ZS, FA, and LE helped in writing, reviewing, and editing.

## Conflicts of Interest

The authors declare that they have no conflicts of interest to disclose.
